# Cost-Consequence Analysis of Mobile Stroke Units vs. Standard Prehospital Care and Transport

**DOI:** 10.3389/fneur.2019.01422

**Published:** 2020-02-12

**Authors:** Andrew P. Reimer, Atif Zafar, Fredric M. Hustey, Damon Kralovic, Andrew N. Russman, Ken Uchino, Muhammad S. Hussain, Belinda L. Udeh

**Affiliations:** ^1^Critical Care Transport Team, Cleveland Clinic, Cleveland, OH, United States; ^2^Frances Payne Bolton School of Nursing, Case Western Reserve University, Cleveland, OH, United States; ^3^Cerebrovascular Center, Cleveland Clinic, Cleveland, OH, United States; ^4^Department of Quantitative Health Sciences, Lerner Research Institute, Cleveland Clinic, Cleveland, OH, United States; ^5^Neurological Institute Center for Outcomes Research, Neurological Institute, Cleveland Clinic, Cleveland, OH, United States

**Keywords:** mobile stroke treatment unit, prehospital emergency care, stroke, cost analysis, transportation of patients

## Abstract

**Background:** Mobile stroke units (MSUs) are the latest approach to improving time-sensitive stroke care delivery. Currently, there are no published studies looking at the expanded value of the MSU to diagnose and transport patients to the closest most appropriate facility. The purpose of this paper is to perform a cost consequence analysis of standard transport (ST) vs. MSU.

**Methods and Results:** A cost consequence analysis was undertaken within a decision framework to compare the incremental cost of care for patients with confirmed stroke that were served by the MSU vs. their simulated care had they been served by standard emergency medical services between July 2014 and October 2015. At baseline values, the incremental cost between MSU and ST was $70,613 ($856,482 vs. $785,869) for 355 patient transports. The MSU avoided 76 secondary interhospital transfers and 76 emergency department (ED) encounters. Sensitivity analysis identified six variables that had measurable impact on the model's variability and a threshold value at which MSU becomes the optimal strategy: number of stroke patients (>391), probability of requiring transfer to a comprehensive stroke center (CSC, >0.52), annual cost of MSU operations (<$696,053), cost of air transfer (>$8,841), probability initial receiving hospital is a CSC (<0.32), and probability of ischemic stroke with ST (<0.76).

**Conclusions:** MSUs can avert significant costs in the administration of stroke care once optimal thresholds are achieved. A comprehensive cost-effectiveness analysis is required to determine not just the operational value of an MSU but also its clinical value to patients and the society.

## Introduction

Delivering effective and timely care for patients experiencing stroke requires extensive and costly resources that are consolidated at comprehensive stroke centers (CSCs). However, many patients do not live in the primary catchment area of a CSC and may present first to a primary stroke center (PSC) or a less capable facility. For those patients requiring comprehensive stroke care, the need for additional transfer to a CSC can result in substantial delays in the delivery of definitive treatment, ultimately leading to poorer outcomes and increased cost (1–3).

Mobile stroke units (MSUs) are the latest approach to improving time-sensitive stroke care delivery (4). MSUs are prehospital ambulances that are fully equipped to perform patient assessments and diagnostic testing (point-of-care lab testing and computerized tomography/angiography) that are necessary to diagnose and initiate stroke treatment prior to transporting to the closest most appropriate facility. The ability of MSUs to provide full diagnostic and initial treatment capabilities changes the paradigm of stroke patient management. Early inquiries have suggested that MSUs can be a cost-effective platform to deliver early stroke therapy (5–7). Previous studies have focused on the clinical benefits from earlier intervention provided by an MSU, mainly through the early administration of a tissue plasminogen activator (tPA) (8). There has been limited inquiry into the utility of MSUs to improve prehospital triage and transfer patients to an appropriate clinical destination, ultimately reducing interhospital transfers (9).

The ability to triage and transport to the correct facility from initial patient contact not only may save time but also can ensure that stroke centers are treating appropriate patients and avoiding the need for secondary interhospital transfers (10). In addition, accurate triage and diagnosis prior to CSC arrival can negate the necessity for the transported patient to be admitted via the emergency department (ED). Patients can be transported directly to the inpatient department, potentially avoiding test duplication and unnecessary use of ED services. Debate continues as to whether the high cost of operating an MSU makes them a financially feasible service. The purpose of this paper is to perform a cost-consequence analysis of MSUs vs. ST including the value of MSUs in identifying and transferring patients to a CSC who require CSC level care, therefore eliminating an emergency department admission and secondary interhospital medical transport.

## Materials and Methods

A comprehensive description of the Cleveland Clinic Mobile Stroke Treatment Unit (MSU) program has been published previously (11). In this paper, a cost-consequence analysis was undertaken within a decision framework incorporating cost and population variability. The model compared the incremental cost of patients served by the Cleveland Clinic MSU vs. their simulated care had they been served by the standard emergency medical service [herein referred to as standard transport (ST)]. This study was approved as secondary medical record review and waiver of consent was granted by the Cleveland Clinic Institutional Review Board (#16-444).

### Target Population

All patients with suspected stroke who had been served by the Cleveland Clinic MSU between July 2014 and October 2015 were identified and used as the model's simulation population. Then, to develop clinical probabilities of each subgroup (ischemic, hemorrhagic), only patients with suspected stroke after initial assessment by the telemedicine neurologist were included. The analysis was conducted from the hospital perspective and included all events from the receipt of an emergency call to the point of patient admission to the hospital. Therefore, clinical probabilities were derived directly from the local patient population served by the MSU representing actual practice and a range of operational costs of the MSUs that are typically owned and operated by health systems—representing additional cost when compared to ST that is provided by local municipalities.

### Model Structure

**Figure 1:**
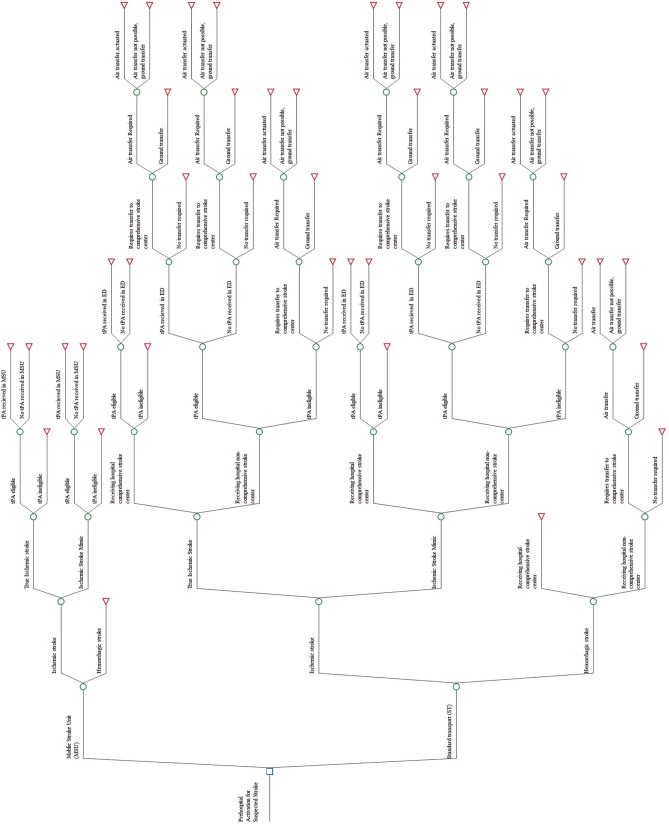
Skeleton decision tree.

The model was developed using TreeAge Pro Suite (TreeAge Software, Inc., Williamstown, MA) with an outline presented in Figure 1. In accordance with published decision analytic methods (12), the model includes all relevant care and event possibilities. The care possibilities of this model include stroke classification; eligible for thrombolysis; recipient of thrombolysis; need to be transferred to a CSC; and whether the transfer was via air or ground. The care possibilities are mutually exclusive (the patient can only follow one path at any one point in time) and exhaustive (all care pathways are included for every patient simulated in the model). Patients can move through the model according to the probabilities of that care or event occurring at that possibility point. For example, in Figure 1, if a patient was following the MSU intervention arm, the first possibility is that he or she may be suffering from an ischemic or a hemorrhagic stroke. For a patient that is suffering from an ischemic stroke, the next possibility is whether he or she is tPA eligible or not. At the end of each possible pathway that a patient may follow, the cost and outcome of the patient reaching that end-point are incorporated.

In order to ensure comparison of similar populations for the MSU intervention arm and the ST intervention arm, the same population was used for both interventions. Model inputs for the MSU intervention were taken from primary MSU data. For the ST intervention, the care of the MSU population was simulated, representing the costs and outcomes had they received care from ST instead of MSU.

### Model Inputs

#### Probabilities and Clinical Data

The clinical probabilities for the MSU arm of this study were abstracted from the identified patient cohort. For the ST arm, the patient cohort pickup location was mapped. Had they been picked up by the MSU, their initial receiving hospital would be a CSC. From the map, it was determined if the patient would have gone to a closer non-CSC hospital had they been served by ST based on local emergency medical services (EMS) transport protocols that dictate transferring suspected strokes to the closest appropriate facility—being either a PSC or CSC. For all patients whose initial receiving hospital would not be a CSC, their likelihood of transfer and method of transfer were simulated. This determination was made using current health system stroke transfer protocols and, when necessary, confirmed via review by at least one stroke team neurologist and one medical transport team director on a case-by case basis (expert opinion). We used the primary clinical data from the identified patient cohort for the probabilities of care possibilities with the MSU arm of the model. The probabilities of the care possibilities for the same population had they received ST were simulated using clinical pathways, expert opinion, and published literature. A baseline value and range are included for every probability. Table 1 includes the value and the range of each probability used in the model.

**Table 1:** Probability and clinical variables, baseline values, and ranges used in the model.

**Variable**	**Baseline value (Range)**	**References**
MSU annual census—suspected/confirmed strokes	355 (100, 600)	MSU data
Probability of ischemic stroke MSU	0.53 (0.43, 0.63)	MSU data
Probability of ischemic stroke ST	0.53 (0.43, 0.63)	MSU data, Expert opinion
Probability of ischemic stroke Mimic	0.27 (0.02, 0.31)	MSU data
Probability tPA eligible MSU	0.82 (0.72, 0.92)	MSU data
Probability tPA eligible ST	0.75 (0.65, 0.85)	MSU data, Expert Opinion
Probability tPA received MSU	0.95 (0.89, 0.98)	MSU data, Expert Opinion
Probability tPA received ED (ST)	0.95 (0.9, 0.99)	MSU data, Expert Opinion
Probability receiving hospital a comprehensive stroke center	0.42 (0.36, 0.56)	MSU data
Probability of requiring interhospital transfer—ischemic stroke	0.41 (0.13, 0.68)	Health system transfer protocol, Expert opinion, (13)
Probability of requiring interhospital transfer—hemorrhagic stroke	0.8 (0.6, 0.9)	Health system transfer protocol, Expert opinion, (14)
Probability of requiring interhospital transfer—Mimic	0.1(0.01, 0.19)	MSU data, Expert Opinion
Probability of requiring transfer by air—ischemic stroke	0.5 (0.4, 0.6)	MSU data, Health system transfer protocol, (15)
Probability of requiring transfer by air—hemorrhagic stroke	0.98 (0.95. 0.99)	MSU data, Health system data
Probability air transfer feasible	0.95 (0.8, 0.99)	MSU data, Health system transfer data

*MSU, mobile stroke unit; ST, standard transport; tPA, tissue plasminogen activator; ED, Emergency Department*.

#### Cost Data

To ensure generalizability beyond our health system, the value of health resources was sourced from credible publicly available sources including reported Medicare reimbursement rates, published hospital costs, and national wage averages reported by the Bureau of Labor and Statistics. Only if data from these sources were not available did we use values reported in peer-reviewed published literature. The annual operating cost for an MSU varied significantly between sources in part because of the location of the MSUs and what staffing model they adopted. To account for this variability, a large range was applied skewed to the more commonly reported lower end of the range. Initial capital was not included but amortization depreciation of MSU and its components was included in the annual cost. The annual costs of ST were not included as it is an existing service and would not require additional funding to continue its service in an area that is also served by an MSU. No drug costs were included as all drugs used on an MSU are assigned to the patient encounter post-transfer and not accounted for in MSU operational costs, and for ST, the drugs are exchanged with the receiving hospital to which the patient was transferred. All costs were adjusted to the same base year, 2017, using the Medicare Component of the Consumer Price Index (CPI)—an index that is specific to healthcare cost and generally greater than the standard national CPI. As the time horizon for this analysis is short, cost discounting was not incorporated. Table 2 includes the baseline value and range of all cost variables used in the model denominated in US dollars.

**Table 2:** Cost variables, baseline value, and ranges used in the economic model.

**Variable**	**Baseline $ (Range)**	**References**
CT scan ^+^	253 (190, 316)	(16)
tPA administration	188 (141,235)	(16)
Observation after tPA	71 (53, 89)	(16)
Emergency department visit—ischemic stroke ^*^	749 (563, 964)	(16, 17)
Emergency department visit—hemorrhagic stroke ^*^	749 (563, 964)	(16, 17)
Telehealth consultation^+^	28 (21, 35)	(16)
Interhospital transfer—air	7,412 (5,559, 9,265)	(16, 18)
Interhospital transfer—ground	723 (542, 904)	(16)
Annual cost MSU operations	600,000 (500,000, 1,200,000)	(5, 7, 19)

*$, US dollars*.

+ *Occur in the MSU prior to transport and hospital admission*.

* *Medicare reimbursement for emergency department billing critical care, independent of other billable costs related to stroke care*.

*CT, computerized tomography; tPA, tissue plasminogen activator*.

#### Model Output (Baseline Analysis)

The model output was the incremental cost between patients with confirmed stroke who had been transferred by an MSU vs. those that were transferred by ST. Any cost differences between the two strategies make up the incremental cost. For example, ST includes the cost of air travel for those patients who went to their closest hospital, but it was not a CSC and they required air transfer. The MSU, however, would have identified these patients as needing a CSC and taken them directly there. The additional cost of air transport would make up part of ST but not MSU, making it part of the incremental cost between the two strategies. All clinical treatments were considered the same for both arms. The timing of treatments, however, differed with the MSU patients receiving a computerized tomography (CT) and tPA (if eligible) in the MSU before reaching the hospital. In accordance with the hospital policy at the Cleveland Clinic main campus, patients brought in by MSU are transferred straight to the neurological unit and not processed through the ED. This process is also followed for patients being transferred from another hospital via Cleveland Clinic's transport team. Because this policy is not standard practice for all CSC's, other hospitals in the Cleveland area that receive patients from the MSU admit the patients to the ED first. Therefore, we account for the additional ED costs of the receiving CSC included for all patients (MSU and ST) in a secondary analysis. Secondary outputs were also tested in the analysis including the number of secondary transfers averted (air and ground) and the number of ED visits averted.

#### Sensitivity Analysis

Uncertainty in the model was analyzed from two aspects: uncertainty in the structure of the model and uncertainty in the variables used within the model. Uncertainty within the model was analyzed using deterministic sensitivity analysis (one-way). This analysis involves propagating through the model, a fixed change (e.g., incorporating a low or high estimate) in the value for each variable, while holding other parameters static. From this analysis, the variables responsible for the greatest change in the model were identified and summarized in a tornado diagram. Variables that had a threshold value, i.e., a value in which the optimal strategy choice changed, were also identified. To address differing hospital policies on the route of admission for transferred stroke patients, sensitivity analysis was also conducted to include the costs of an admission via the ED as opposed to the baseline analysis where patients are transferred straight to a stroke unit, avoiding an ED admission and its associated costs.

To test the uncertainty in the values of the variables used in the model, probabilistic sensitivity analysis was conducted. This form of analysis allows the uncertainty in the estimates of all the variables in the model to be assessed simultaneously. For each iteration, the value input for any variable is sampled from the distributions (e.g., gamma distribution for costs), specified for each of the variables included in the model. For this study, 10,000 iterations were undertaken. The results determined the range of plausible incremental costs and the confidence intervals of these different incremental costs between the MSU and ST occurring. From these results, a cost-effectiveness acceptability curve was generated. This curve displays what is the probability of the plausible incremental costs between the two strategies.

## Results

### MSU Data Results

**Figure 2:**
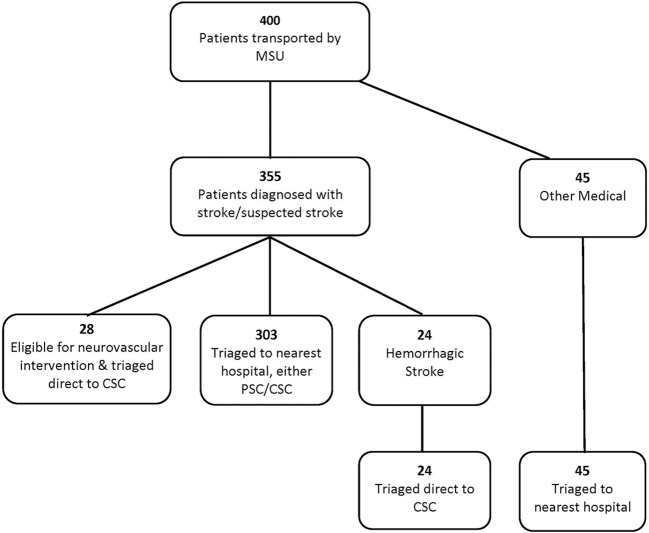
Patient flow diagram.

During the study period, 400 patients were transported by the MSU; 54% of the patients were female; the median age was 64 years (SD 16.6, range 17–104). At the time of the MSU encounter, 331/400 (83%) patients were diagnosed with stroke or suspected ischemic stroke, 24/400 were diagnosed with hemorrhagic stroke, and 45/400 cases were other medical cases—cases with non-stroke etiology (e.g., overdose, hypoglycemia). Fifty-two of the cases required a CSC, with 28/52 being eligible for neurovascular intervention and the remaining were hemorrhagic stroke cases (Figure 2).

### Baseline Value Analysis Results

As anticipated, the ST pathway cost less over 1 year than the MSU pathway as the MSU pathway includes the costs of running the MSU including labor and maintenance. At baseline values, the cost of ST was $785,869 and the cost of MSU $856,482. The incremental cost of the MSU vs. ST was $70,613, indicating that to treat the same patient population, the MSU would cost an additional $70,613. The MSU avoided 76 secondary transfers and 76 ED encounters.

### Sensitivity Analysis

A tornado analysis was performed for all the variables within the model. This analysis identifies the variables that are the greatest drivers of outcome variability as compared to the baseline expected value (EV). From this analysis, six variables were identified as being the greatest drivers. In decreasing impact, the variables were “Number of stroke patients,” “Probability of requiring transfer to a CSC,” “Annual cost of MSU operations,” “Cost of air transfer,” “Probability initial receiving hospital is a CSC,” and “Probability of ischemic stroke with standard transport.” These identified variables all had an identified threshold value at which the choice of optimal strategy would change, i.e., MSU would become more cost effective than ST and therefore be the strategy of choice. MSU would be the choice strategy if any of these thresholds were met with all other variables remaining the same: The number of stroke patients seen was >391; if the probability of requiring transfer to a CSC with ischemic stroke (IS) was >0.52; if the MSU annual operating costs were below $696,053; the cost of air transfer was >$8,841; the probability that the initial receiving hospital is a CSC is <0.32; or of the probability of IS with ST was <0.76. All remaining variables had negligible impact on the variability of results and did not have a threshold value at which MSU would become cheaper than ST.

It is not standard policy for all CSCs to accept a direct admission from the MSU or of a transferred stroke patient, with most still requiring an ED encounter and evaluation. To address this variance, the analysis was also run with the inclusion of an ED visit for all MSU patients. At the baseline values, the same number of secondary transfers was averted. However, the cost for ST for 355 patients remained at $785,869. The cost of the MSU increased to $1,122,199. The additional cost of the MSU as compared to ST increased to $336,331, more than four times the additional cost if MSU patients are admitted directly to the unit, bypassing the ED. Without a direct admit policy, the MSU would need to treat 632 to be cost neutral.

**Figure 3:**
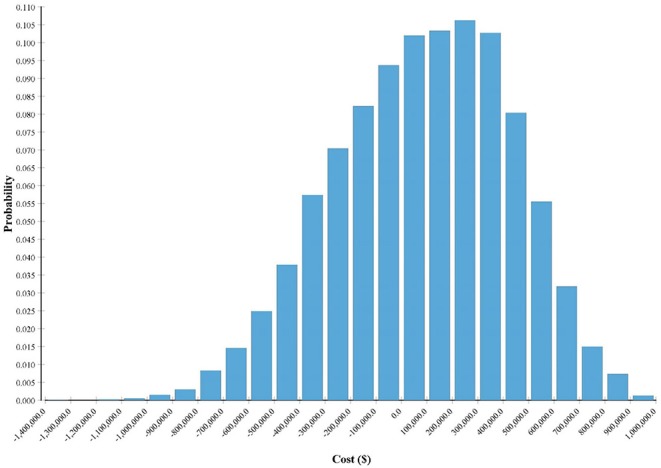
Monte Carlo probability distribution of MSU point of cost neutrality.

Monte Carlo probability sensitivity analysis was conducted for 10,000 iterations. For each iteration, the value of each variable was randomly selected according to each variable assigned distribution. The median was close to the baseline analysis at $81,882, but the results were positively skewed (an MSU costs greater than ST) with a large spread (Figure 3). The results indicated that for 60.3% of the iterations, ST was the choice (i.e., cheaper strategy) and an MSU was the choice strategy for the remaining 39.7% of the iterations. Results further indicate that the cost of ST is far more variable than that of an MSU. For example, the difference in cost between the 10th percentile and the 90th percentile for an MSU was ~$425,000. The same cost difference for ST was more than double at ~$915,000. Full probabilistic sensitivity analysis results are presented in Table 3.

**Table 3:** Monte carlo probability distribution statistics (*n* = 10,000).

**Statistic**	**MSU $**	**ST $**	**Incremental cost $ (MSU-ST)**
Mean	855,507	772,969	82,538
Median	832,036	750,154	81,882
Standard deviation	159,138	346,128	−183,157
SQRT (variance/size)	1,591	3,423	−1,832
Minimum	533,138	153,391	379,747
10th percentile	664,155	326,856	337,299
90th percentile	1,090,941	1,239,733	−148,792
Maximum	1,346,246	2,079,937	−733,692

*$, US dollars*.

## Discussion

MSUs require a significant expense on capital and significant ongoing operating costs. Understanding an MSU's impact on not just clinical outcomes but also resource utilization can help guide decision makers on the value of this intervention for the population in question. Our primary finding of minimal increased costs (~$70 K) while avoiding secondary interhospital transfers and ED admissions provides additional financial and health services utilization considerations in the discussion of cost-efficiency related to MSUs. While most cost considerations revolve around startup and maintenance cost (20), all financial impacts need to be incorporated. From a healthcare delivery and efficiency perspective, avoiding emergency department admissions and secondary interhospital transfers (often by helicopter with the average cost of $7,000+/flight) (16, 18) can contribute to significant savings on an annual basis without adversely affecting clinical outcomes. In our case, the cost savings realized by just avoiding the secondary interhospital transfer can account for ~35% of the annual operating cost of the MSU. Additionally, because our MSU bypasses the ED and admits the patient directly to the treating inpatient bed, additional cost savings are realized.

MSUs' cost-effectiveness may become more pronounced as health systems incorporate their use in the ongoing development of stroke systems of care (21–24). As previously discussed, the ability of MSUs to accurately diagnose patients and transport them to the appropriate level of care is a major benefit. Ultra-early tPA administration may in itself reduce morbidity and mortality and thus result in costs savings during the initial hospital stay through rehabilitation (25). Similar cost savings may also be realized via early diagnosis in the MSU to identify candidates for mechanical thrombectomy and transfer the patient directly to intervention as well (26).

As MSUs are a modern technology and the discussion nationally of how to bill for telemedicine continues to evolve, we assumed that none of the aspects of the MSU encounter were reimbursable by a health insurance system. The next study planned will be to conduct this analysis modeling for the impact of potentially billable aspects. Of priority importance is the billing of the actual transport by an MSU. As this is a topic of discussion nationally, evidence needs to be provided to payers—both public and private—that reimbursing MSU transport at the same rate that ground critical care ambulances bill would be both cost-effective and allow a greater implementation of this service. Our results provide support that reimbursing MSUs at critical care ambulance rates (Medicare critical care reimbursement $690 vs. $403 for standard 911 billing), a necessary component of achieving cost-neutrality, would significantly reduce an MSU's operational costs, increase the efficiency of stroke care delivery, and conserve scarce health system and transport resources.

Previous cost-effectiveness analyses have identified that, with optimal staffing, an MSU can be cost-effective even in less dense population centers (5, 27). Our MSU has implemented and demonstrated the efficacy of using telemedicine to support efficient operation of the MSU (11). The immediate benefit of using telemedicine for the stroke neurologist's evaluation and clinical disposition enables the stroke neurologists to review all patient-related medical records, including the current CT scans and previous history and imaging, predict the patient's required level of care ensuring that the patient is transported to the appropriate stroke center, and mobilize the necessary resources (i.e., MRI or intervention suite) prior to the patient's arrival.

As our study shows, the operating costs of an MSU vary considerably with changes in the number of stroke patients seen annually, probability of requiring transfer to a CSC with IS, annual cost of MSU operations, cost of air transfer, and probability of ischemic stroke with ST. For all of these variables, they had a threshold at which an MSU would be more cost-effective, while all other variables remained the same. These are significant findings that can be used as a decision aid to determine not only if an MSU service should be initiated but also what geographical area it should serve. For example, operating an MSU in a geographic area close to a CSC would be an inefficient use of resources as the patients most likely would have been transported to the CSC by ST, with little to no time savings.

This pilot study presents the initial development and results of a model to support cost-effectiveness efforts related to an MSU. This model captures only a piece of a complex intervention that is highly dependent on many different factors. There are several limitations of this analysis that need to be addressed in future research. All costs are analyzed using United States dollars. The probabilities used in the modeling (e.g., probability of ischemic stroke, eligible for tPA, require interhospital transfer) were based on our local data and small sample size and should be adjusted to local population characteristics. The annual cost of MSU operations had significant variability among sources and depending greatly on how each MSU is set up and staffed. While the operating cost accounts for dry runs or transfer of patients who were found not to be stroke patients, we did not include the cost to ST of arriving at the scene of a suspected stroke patient but having the MSU transfer the patient. The justification for this exclusion is that while ST had no patient transfer and therefore no billable event, the vehicle was able to return much more quickly to regular service and be available for other calls.

Another limiting factor of this analysis is the model assumed a high frequency of air transfer based on our health systems rates. Systems within a large metropolitan area or with limited air transfer capabilities may use less costly ground transfer more commonly. As averting air transfer is a significant part of cost savings attributable to an MSU, this could have a significant impact on the decision to use an MSU. While the modeling approaches provide a robust approach that incorporates variability around each probability used and our probabilities are within the range of other reports (16, 28), it is broad to ensure it is generalizable. However, owing to the differences between different populations and locations and the sensitivity of this analysis to these changes, local values should be used to ensure relevance to a specific population and location (5). This analysis demonstrates scenarios in which an MSU may be financially viable. It is, however, very conservative in the potential benefits of the MSU. We assumed no billable items for the MSU in this analysis. As MSUs and telemedicine become more mainstream, it is anticipated that many of an MSU's activities may become billable items, therefore increasing its financial viability. More importantly, this study looks purely at operations and does not include any clinical outcomes. With the results of ongoing clinical studies into the clinical benefit of an MSU becoming available, these benefits could be of significant value to the hospital, the patient, and the society. For example, if the time saving from an MSU can avoid or at least avert disability in a stroke patient, reduced medical costs, reduced caregiver costs, reduced disability support, etc., would all be attributable to the value of an MSU. As data on MSUs continue to be collected, this study can serve as a base for a much more comprehensive study where all resource and clinical implications, especially long-term quality of life, are included.

## Conclusion

MSUs can avert significant costs in the administration of stroke care once optimal thresholds are achieved. Cost savings realized by avoiding secondary interhospital transfers can account for ~35% of the annual operating cost of the MSU. It also demonstrates that the budgetary impact of an MSU within a health system can vary significantly by clinical and geographic factors, which should be considered in the decision to set up and run an MSU service. A comprehensive cost-effectiveness analysis is required to determine the clinical and operational value of an MSU.

## Data Availability Statement

The datasets generated for this study will not be made publicly available as regulated by the IRB. Requests to access the datasets should be directed to Andrew P. Reimer, reimera@ccf.org.

## Ethics Statement

The studies involving human participants were reviewed and approved by Cleveland Clinic IRB. Written informed consent for participation was not required for this study in accordance with the national legislation and the institutional requirements.

## Author Contributions

APR, AZ, KU, and BU contributed to study conception and design. APR and AZ organized the data. BU performed the cost consequence analysis. APR and BU wrote the first draft of the manuscript. FH, DK, ANR, and MH contributed to interpreting study results, drafting and critical revision, read and approved the submitted version.

### Conflict of Interest

The authors declare that the research was conducted in the absence of any commercial or financial relationships that could be construed as a potential conflict of interest.
